# Local walking and cycling by residents living near urban motorways: cross-sectional analysis

**DOI:** 10.1186/s12889-019-7621-4

**Published:** 2019-11-01

**Authors:** Eleanor F. J. Powers, Jenna Panter, David Ogilvie, Louise Foley

**Affiliations:** 1 MRC Epidemiology Unit & UKCRC Centre for Diet and Activity Research (CEDAR), School of Clinical Medicine, University of Cambridge, Box 285 Institute of Metabolic Science, Cambridge Biomedical Campus, Cambridge, CB2 0QQ UK; 2 Health Education East of England, 2-4 Victoria House, Capital Park, Fulbourn, Cambridge, CB21 5XB UK

**Keywords:** Active travel, Roads, Motorway, Walking, Cycling, Severance, Infrastructure

## Abstract

**Background:**

Everyday activities, such as walking or cycling, may be a feasible and practical way to integrate physical activity into everyday life. Walking and cycling for transport or recreation in the area local to a person’s home may have additional benefits. However, urban planning tends to prioritise car use over active modes. We explored the cross-sectional association between living near an urban motorway and local walking and cycling.

**Methods:**

In 2013, residents living in an area (a) near a new urban motorway (M74), (b) near a longstanding urban motorway (M8), or (c) without a motorway, in Glasgow, Scotland, were invited to complete postal surveys assessing local walking and cycling journeys and socio-demographic characteristics. Using adjusted regression models, we assessed the association between motorway proximity and self-reported local walking and cycling, as well as the count of types of destination accessed. We stratified our analyses according to study area.

**Results:**

One thousand three hundred forty-three residents (57% female; mean age: 54 years; SD: 16 years) returned questionnaires. There was no overall association between living near an urban motorway and the likelihood of local walking or cycling, or the number of types of local destination accessed by foot or bicycle.

In stratified analyses, for those living in the area around the new M74 motorway, increasing residential proximity to the motorway was associated with lower likelihood of local recreational walking and cycling (OR 0.63, 95% CI: 0.39 to 1.00) a pattern not found in the area with the longstanding M8 motorway. In the area near the M8 motorway residential proximity was statistically significantly (*p* = 0.014) associated with a 12% decrease in the number of types of destination accessed, a pattern not found in the M74 study area.

**Conclusions:**

Our findings suggest that associations between living near a motorway and local walking and cycling behaviour may vary by the characteristics of the motorway, and by whether the behaviour is for travel or recreation. The lack of associations seen in the study area with no motorway suggests a threshold effect whereby beyond a certain distance from a motorway, additional distance makes no difference.

## Background

A third of adults (~ 1.5 billion individuals) do not meet World Health Organization (WHO) recommendations for physical activity [1]. Physical inactivity accounts for approximately 9% of premature mortality globally [2], and is linked with physical and mental ill health [2]. Any increase in physical activity is beneficial to health [3], and everyday activities, such as walking or cycling for transport or recreation [4, 5] may be a feasible and practical way for populations to integrate physical activity into everyday life [5–7].

Walking and cycling in the neighbourhood local to one’s home, either as active travel or for recreation, may have additional benefits in developing local social networks and social capital [8, 9], assets independently associated with increased local active travel [10–12], and other aspects of health and wellbeing [13, 14], suggesting wider social benefits. Reducing car use is a current policy priority on equity, health and environmental grounds [15]. However, urban planning has historically emphasised motorised transport, especially private cars, potentially to the detriment of walking and cycling [16, 17].

Controlled-access highways (motorways or freeways), do not permit any use for non-motorised travel and have specific, controlled exit and entry points [18]. In past planning disputes, their hypothesised impacts when built in urban settings have been controversial, being argued on one hand to impede local walking and cycling via community severance (the separation of residents from local amenities and social networks [19]) but on the other to facilitate local walking and cycling via the removal of traffic from local roads to leave them quieter and safer [20, 21]. Systematic reviews indicate that new major roads in urban areas increase noise and severance [22], and postulate a relationship between roads and diminished physical activity [8], but empirical evidence is sparse. Very few studies have examined the relationship between the presence of major road infrastructure in residential areas and walking or cycling. Social and physical environments can influence physical activity behaviour [23], so it is reasonable to hypothesise that the presence of major roads in urban settings has some association with local walking and cycling. Although behavioural influences on recreational walking and cycling may differ from those on active travel [24], aesthetics are considered an important determinant of the former, which may be harmed by road infrastructure and road traffic [24, 25].

In Glasgow, Scotland, the extension of the M74 motorway across a predominantly urban, deprived portion of the city commenced in 2008, and was completed in 2011 at a final cost of approximately £800 million [26]. This presented an opportunity to examine the effects of this new urban motorway on behaviour and health in local residents using a natural experimental study. Increasingly, natural experiments are recognised as key evidence in such situations where randomisation is impossible [27]. A previous analysis of total travel behaviour by all modes via 1 day diaries indicated that the new motorway promoted total travel, and specifically car use in those living nearby, but no effects (either positive or negative) were found on overall levels of active travel [20]. In this paper we focus on a distinct set of survey questions that we used to address active travel specifically within respondent’s local home neighbourhood, and over the past 7 days. We examine active travel to, and recreational use of, amenities (including public transport hubs) and social networks within the neighbourhood, exploring the cross-sectional association between living near an urban motorway and local walking and cycling.

## Methods

### Design

This cross-sectional analysis uses follow-up data from a larger longitudinal natural experimental study. This study recruited subjects before M74 motorway construction (2005) and again 2 years after the M74 opened (2013); however the set of survey questions of interest to this paper were added in 2013, and not recorded at the first time point.

The study received ethical approval from the University of Glasgow (baseline reference FM01304; follow-up reference 400,120,077). Further information on the baseline study hypotheses, methods [28] and sample characteristics [29] and other findings from the longitudinal study [26] can be found elsewhere.

### Patient and public involvement

The study design was shaped by a formative programme of community engagement with organisations representing and serving local communities. This is described in detail in the final study report [26].

### Setting, sampling and recruitment

The setting of the study was Glasgow, the fourth largest city in the UK (593,200 inhabitants) [30], with the lowest life-expectancy in the UK [31], and characterised by extremes of affluence and deprivation [32].

**Fig. 1:**
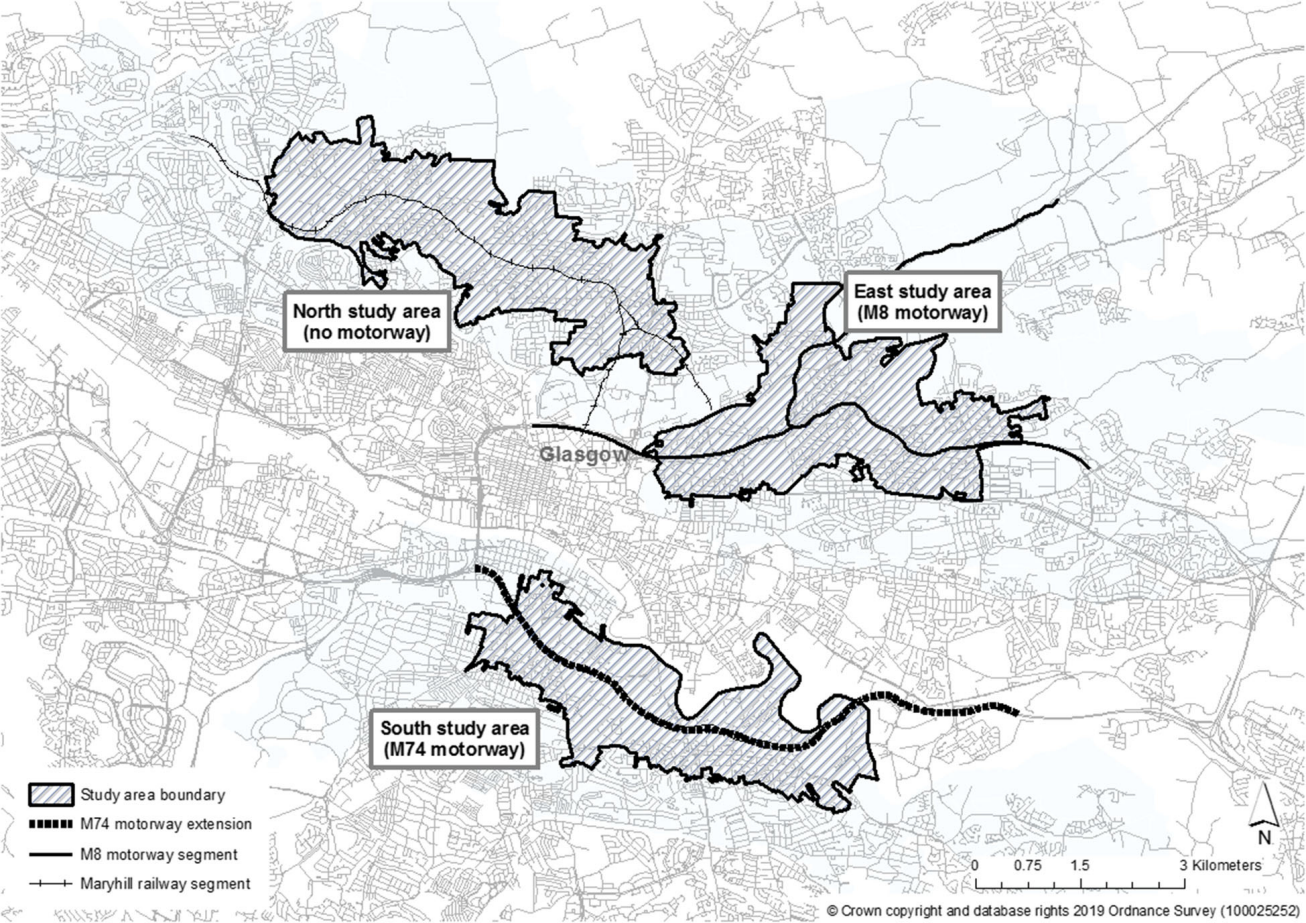
Study areas. Reproduced with permission from *Ogilvie* et al*, Health impacts of the M74 urban motorway extension: a mixed-method natural experimental study, Public Health Research, 2017, 5(3)* (p.10) [26]© Queen’s Printer and Controller of HMSO 2017.© Crown copyright and database rights (2019) Ordnance Survey (100025252)

In 2013, 9000 surveys were sent to randomly selected addresses from the Royal Mail Postcode Address File from one of the three study areas; an area surrounding the new (M74) motorway, an area surrounding an existing motorway (the M8, constructed in the 1960s) and an area with a railway but no motorway infrastructure [28]. The delineation of these areas has been well described [20, 26, 28]; in brief, having defined a meaningful ‘intervention area’ around the proposed M74 corridor, a combination of field visits and aggregate transport infrastructure and census data including levels of unemployment and deprivation, car and home ownership and chronic illness were used and summarised for this area. Suitable locations for the two potential reference areas were then identified, ensuring that the characteristics of the intervention and these areas were broadly similar based on the above listed socioeconomic characteristics, as well as topographical and urban morphological features such as street network density. The areas are shown in Fig. 1. All baseline participants with current contact details and who had agreed to be re-contacted were mailed a survey. Adults aged 16 or over and those with the next birthday were invited to respond and were pre-notified via post before having questionnaires sent for self-completion. A £5 voucher incentive was offered, and one follow-up reminder letter sent. Participants replying within approximately 3 months of the original mailing were included in the study [26]. The full survey has been published elsewhere and can be accessed in the final study report [26].

### Assessment of motorway exposure

Exposure was objectively defined using the GIS as the straight-line distance from each participant’s home address to the nearest motorway infrastructure, weighted via transformation with the negative natural log (higher values indicate greater proximity). A unit change in exposure might correspond, for example, to the difference between living 100 m or 300 m from the motorway, or between those living 300 m to 800 m away. This transformation has been undertaken in previous analysis of this dataset, and addresses the concept that a unit increase in proximity is likely to have a non-linear effect as initial proximity increases [20]. Straight line distance to any part of the motorway was felt to be the most relevant quantification of exposure, as any effects on walkability - severing or aesthetic - would be encountered at any part of the infrastructure. Sensitivity analysis with road network distance as an alternative quantification of exposure, as later described, further explored this issue.

### Assessment of local walking and cycling outcomes

Within the survey, participants indicated whether they had accessed any of the following ten local neighbourhood destinations by foot or bicycle:

- Local shop (e.g. grocery shop, bakery, butcher)
- Supermarket
- Local services (e.g. bank, cash machine, post office, chemist, library)
- Restaurant, café, pub or bar
- Fast-food restaurant or takeaway
- Bus stop, tram, train or underground station
- Sport and leisure facility (e.g. swimming pool, sports field or fitness centre)
- Open recreation area (park or other open space)
- Family or friend’s house
- Work, school or training institute

Participants were instructed in the question wording that ‘your local area is everywhere within a ten-minute walk (about half a mile) from your home’.

For each destination, there were three options: ‘Walked here in the past 7 days’; ‘Cycled here in the past 7 days’; or ‘Did not walk or cycle here’. Additionally, participants reported whether they had walked or cycled for recreation in their local area in the past 7 days. Again, there were three response options: ‘Walked for recreation in the past 7 days’; ‘Cycled for recreation in the past 7 days’; or ‘Did not walk or cycle for recreation’. For analysis, we generated three binary (yes or no) outcomes and two count outcomes. We did not impute missing outcomes. The binary outcomes were:

- Local walking and cycling for transport (to neighbourhood destinations, excluding recreation) – dichotomised to none/some
- Local walking and cycling for recreation (using the question on recreation only) – dichotomised to none/some
- Local walking and cycling of any type (for transport or recreation) - dichotomised to none/some

The count outcomes were:

- Number of local destinations accessed, including using the local area as a destination for recreation (range 0–11) – dichotomised to below or above the median
- Number of local destinations accessed, excluding recreational use of local area (range 0–10) dichotomised to below or above the median

### Assessment of covariates

In the survey, participants self-reported information on several covariates theorised to confound the association between living near a motorway and local walking and cycling. These were:

- Age
- Gender
- Home ownership, dichotomised to owns house (including with a mortgage) versus other
- Regular activities outside the home, dichotomised to activities outside home (e.g. working, studying or volunteering) versus other
- Car ownership, dichotomised to owns a car versus does not own a car
- Self-reported difficulty walking (yes or no)
- Years lived in the local area

### Analysis

Each of the three study areas were contextually different and so we conducted all descriptive and maximally-adjusted analyses separately for each study area. We compared the demographic characteristics of participants who did and did not report walking and cycling locally, and who reported accessing above and below median number of destinations. Only those participants with complete data on outcomes, exposures and covariates were included in the analysis of the maximally adjusted models.

We assessed the relationship between motorway proximity and walking and cycling using logistic regression. We assessed the relationship between motorway proximity and destinations accessed using negative binomial regression, having trialled a Poisson model and found it to fit poorly. In both analyses, Model 1 was unadjusted, Model 2 adjusted for age and sex, Model 3 as Model 2 with the addition of home-ownership, and the final Model 4 as Model 3 with the addition of regular activities outside the home, car ownership, difficulty walking and years lived in the local area.

If participants replaced some local active journeys, for example grocery shopping, with motorway journeys in cars, then road network distance from residence to the motorway would be a more meaningful exposure than straight-line distance. Therefore, we conducted a sensitivity analysis, using an alternative definition of exposure which was calculated using the negative natural log transformed road-network distance from residence to the nearest motorway access point.

## Results

### Response and demographic characteristics

One thousand three hundred forty-three individuals (16% response rate) responded to the postal survey in 2013. Of these, 1276 (95% of respondents) had some outcome and complete covariate information and were included in the maximally adjusted negative binomial regression. They had a mean age of 54 years (standard deviation [SD] 16 years) and 43.5% were male. No statistically significant differences were observed in characteristics of the participants across the three study areas, apart from their exposure to motorway infrastructure (Table 1). For the logistic regression analysis, those who had indicated no walking or cycling in some items but had not completed all items were excluded (*n* = 13) and so the maximally adjusted logistic regression contained 1263 participants (94% of respondents).

**Table 1:** Characteristics of the total sample for participants with some outcome and complete covariate data, and by study area

Characteristics^a^	Overall (*n* = 1276)	South (new motorway) (*n* = 420)	East (longstanding motorway) (*n* = 416)	North (no motorway) (*n* = 440)
Median distance to nearest motorway infrastructure in metres (IQR)	512 (1813)*	414 (238)	349 (270)	2803 (1452)
Mean age in years (SD)	54.0 (16.0)	53.5 (16.2)	53.4 (16.3)	55.0 (15.4)
Male (%)	555 (43.5)	197 (47.0)	174 (41.8)	184 (41.8)
Full home ownership (%)	683 (53.5)	224 (53.3)	221 (53.1)	238 (54.1)
Activities outside home (work, student or volunteers) (%)	625 (49.0)	212 (50.5)	206 (49.5)	207 (47.1)
Not owning a car (%)	563 (44.1)	191 (45.5)	189 (45.4)	183 (41.6)
Difficulty walking (%)	350 (27.4)	108 (25.7)	116 (27.9)	126 (28.6)
Median years lived in local area (IQR)	15.3 (23.3)	13.1 (20)	19.5 (24.7)	16.3 (24.8)

*IQR* Interquartile range, *SD* Standard deviation

* *p* = < 0.01 difference across groups

^a^Reported as: number of participants (%) except where otherwise specified

### Descriptive analysis of local walking and cycling

Of the 1263 participants with complete covariate and outcome data, included in the maximally adjusted logistic regression analysis, 89% (*n* = 1128) reported having walked or cycled in their local area, either to a destination or for recreation, in the past 7 days. 44% (*n* = 560) indicated recreational walking and/or cycling.

Those reporting no walking or cycling of any type in the past 7 days in their local area were statistically significantly older, more likely to have difficulty walking and more likely not to own a car than those who reported they had walked or cycled, and differed in other covariates (Table 2).

**Table 2:** Characteristics of participants by category on the total walking and cycling binary outcome

Characteristics^a^	Reporting any local walking and cycling in past 7 days (*n* = 1128)	Reporting no local walking and cycling in past 7 days (*n* = 135)
Mean age in years (SD)*	53.1 (15.7)	60.4 (16.5)
Male (%)	491 (43.5)	57 (42.2)
Full home ownership (%)	614 (54.4)	62 (45.9)
Activities outside home (work, study, or volunteer) (%) *	583 (51.7)	38 (28.2)
Not owning a car (%) **	510 (45.2)	48 (35.6)
Difficulty walking (%) *	242 (21.5)	97 (71.9)
Median years lived in local area (IQR)*	15.0 (23.0)	19.0 (30.8)

*IQR* Interquartile range, *SD* Standard deviation

*p value < 0.01

**p value < 0.05

^a^Reported as: number of participants (%) except where otherwise specified

In the 1276 participants with any outcome data and complete covariate data, the median count of destination types accessed, including using the local area for recreation, was 4 (Interquartile range [IQR] 4) ranging from 0 to 11 types accessed. The type of destination most commonly accessed by walking and/or cycling was ‘local shop’, with 933 participants (73%) reporting accessing such a place in the past 7 days. The least commonly accessed was ‘sport facility’, reported by 166 participants (13%). Seven hundred ninety-six participants (64%) had accessed a public transport station by walking and/or cycling in the past 7 days.

Those (*n* = 646) who had accessed less than the median number of places differed significantly from those who had accessed more than the median in several characteristics, similar to the differences between those who did and did not walk at all (Table 3).

**Table 3:** Characteristics of participants, by the count of types of destination accessed outcome (dichotomised to above or below the mean average count of types accessed)

Characteristics^a^	Accessing 0 to 4 destinations by walking and cycling in past 7 days (less than median) (*n* = 646)	Accessing more than 4 destinations by walking and cycling in past 7 days (more than median) (*n* = 630)
Mean age in years (SD)*	55.8 (16.4)	52.2 (15.3)
Male (%)	272 (42.1)	283 (44.9)
Full home ownership (%)	340 (52.6)	343 (54.4)
Activities outside home (work, study, or volunteer) (%) *	293 (45.4)	332 (52.7)
Not owning a car (%) *	255 (39.5)	308 (48.9)
Difficulty walking (%) *	261 (40.4)	89 (14.1)
Median years lived in local area (IQR)**	18.0 (25.9)	14.4 (21.8)

*IQR* Interquartile range, *SD* Standard deviation

*p value < 0.01

**p value < 0.05

^a^Reported as: number of participants (%) except where otherwise specified

### Relationship between motorway proximity and local walking and cycling

In the maximally adjusted logistic regression models, there were no overall associations between motorway exposure and the likelihood of local walking and cycling. However, stratified analyses by study area indicated that for participants in the area around the new motorway (M74) only, proximity to the motorway was associated with a higher likelihood of local walking and cycling for active travel (Odds Ratio [OR] 1.95, 95% Confidence Interval [CI]: 0.95 to 4.02, *p* = 0.070) but a lower likelihood of local walking and cycling for recreation (OR 0.63, 95% CI: 0.39 to 1.00, *p* = 0.050) (Table 4).

**Table 4:** Odds ratio for unit change in proximity to any motorway infrastructure for likelihood having walked and/or cycled in the local area, and by walking and cycling type, by study area, using maximally adjusted model

Study Area	Type of walking and/or cycling in local area		
	Odds ratio (95% CI) for total walking and cycling	Odds ratio (95% CI) for walking and cycling as active travel	Odd ratio (95% CI) for walking and cycling for recreation
South (new motorway)	1.98 (0.93,4.22)	1.95 (0.95,4.02)	0.63 (0.39,1.00)
East (longstanding motorway)	0.89 (0.52,1.52)	0.90 (0.53,1.52)	0.97 (0.68,1.37)
North (no motorway)	1.30 (0.54,3.14)	1.40 (0.58,3.37)	0.70 (0.41,1.20)

Models adjusted for age, gender, home ownership, regular activities outside the home, car ownership, difficulty walking and years lived in the local area

### Relationship between motorway proximity and number of local destinations accessed

In the maximally adjusted negative binomial regression models, there were no overall associations between motorway exposure and number of types of local destinations accessed via walking or cycling, with or without inclusion of accessing the local area itself for recreation. However, in analysis stratified by study area, in the area around the longstanding motorway (M8), a unit increase in proximity to motorway infrastructure was statistically significantly (*p* = 0.014) associated with about a 12% decrease in count of types of destination accessed via active travel (Table 5).

**Table 5:** Negative binomial regression coefficient for unit change in proximity to any motorway infrastructure for count of types of destination accessed, by types of destination and by study area, using maximally adjusted model

Study Area	Type	
	Rate ratio for count of all types of destination accessed by walking and cycling, including use of local area for recreation	Rate ratio of count of types of destination accessed via active travel
South (new motorway)	0.97 (0.86,1.08)	0.98 (0.88,1.09)
East (longstanding motorway)	0.89 (0.80,0.98)	0.88 (0.79,0.97)*
North (no motorway)	0.90 (0.76,1.06)	0.90 (0.76,1.05)

Models adjusted for age, gender, home ownership, regular activities outside the home, car ownership, difficulty walking and years lived in the local area

*p value < 0.05

### Sensitivity analysis

The sensitivity analysis repeated the primary analysis, with the exposure variable altered to road network proximity to nearest motorway access point (transformed via negative natural log). In this analysis, no statistically significant associations were found in the logistic regression or negative binomial regression models overall. This was consistent with the main analysis. In stratified analyses, however, a unit increase in this exposure was associated with higher likelihood of local active travel (OR 2.03, 95% CI: 1.00 to 4.12, *p* = 0.050) in the area around the longstanding motorway (M8) (Table 6). This contrasts with the main analysis, where a similar association was seen for the new motorway (M74) but not for the M8.

**Table 6:** Odds ratio for unit change in road network proximity to motorway access point for odds of total walking and cycling, and walking and cycling by type, by study area

Study Area	Type of walking and cycling in local area		
	Odds ratio (95% CI) for total walking and cycling	Odds ratio (95% CI) for walking and cycling as active travel	Odd ratio (95% CI) for walking and cycling for recreation
South (new motorway)	1.07 (0.49,2.34)	0.96 (0.46,1.98)	0.73 (0.49,1.11)
East (longstanding motorway)	2.02 (0.98,4.16)	2.03 (1.00,4.12)*	1.53 (0.77,1.74)
North (no motorway)	1.23 (0.51,2.97)	1.33 (0.55,3.21)	0.63 (0.36,1.09)

Models adjusted for age, gender, home ownership, regular activities outside the home, car ownership, difficulty walking and years lived in the local area

*p value =0.05

## Discussion

### Main findings

In this cross-sectional analysis, we found no overall association between exposure to urban motorway infrastructure and the likelihood of local walking or cycling, or of the number of local destinations accessed on foot or by bicycle.

However, in the analyses stratified by study area, we found that in the area around the new motorway (M74), a unit increase in proximity to the motorway was associated with a lower likelihood of local recreational walking and cycling (OR 0.63, 95% CI: 0.39 to 1.00, *p* = 0.050) a pattern not found in the area with the longstanding motorway (M8). Conversely, we found that in the area near the longstanding motorway a unit increase in proximity to motorway infrastructure was statistically significantly (*p* = 0.014) associated with about a 12% decrease in count of types of destination accessed via active travel, a pattern not found in the new motorway (M74) study area. Sensitivity analysis results, with an altered exposure definition, also differed by area.

This provides important quantitative evidence that an association between exposure to urban motorway infrastructure and local walking and cycling behaviour exists and suggests it may vary by the characteristics of the motorway to which a participant is proximal, such as the age of the motorway. The conflicting nature of the findings mirror the conflicting prior claims [21] that living near a motorway could have both positive and negative impacts on local walking and cycling. The lack of associations seen in the study area with no motorway suggests a threshold effect whereby beyond a certain distance from a motorway, additional distance makes no difference.

### Strengths and limitations

This study adds to a growing body of evidence examining the association between the built environment and health behaviour and is one of few to address the associations of walking and cycling with the presence of urban major road infrastructure. Using a GIS, we objectively defined exposures, using models controlled for a series of potential confounders.

We also acknowledge the study limitations. In this cross-sectional study, temporality of association between exposure and outcome could not be demonstrated. Because the exposure could not be assigned at random there may also be some residual confounding in unmeasured factors or characteristics [33]. The potential confounding influence of socioeconomic status, which may influence both location of residence and options for travel beyond walking and cycling, is particularly important. We used car and home ownership as proxies of socioeconomic status in the fully adjusted models, however these are blunt measure for this nuanced characteristic. We also derived a measure of whether participants were employed, in education or volunteering (or not) which may have captured both a measure of socioeconomic status, but also in part provided a purpose for travel or reasons to leave the house.

There was a comparatively low response to the survey, which may limit the external validity of the findings, although our response rate was not unusual for this type of natural experimental study [34, 35]. Our examination of likelihood of active travel reports relative impacts from a controlled comparison across three similar study areas, which improves the methodological robustness of the study.

Further biases may be introduced due to the self-report nature of our outcome. However, error introduced during self-report is unlikely to differ by level of exposure to motorway infrastructure. Our self-report tool was a relatively blunt indicator of local walking and cycling and did not assess duration of activity. Similarly, the count of types of destinations accessed should not be misinterpreted as number of trips made to particular destinations, or reflective of the variety of destinations of that type in the area. For example, a person making one journey in the 7 days to one shop would be recorded as the same as a person making daily journeys to seven different shops during the study period.

In an effect termed ‘resident/migrant bias’ or ‘residential self-selection’ [4], individuals may choose to live in areas which reflect their pre-existing lifestyle choices. In this example, someone who does not intend to walk or cycle locally might move to an area where it is not easy to walk and cycle because it does not matter to them, or a person intending to travel predominantly by car move near a motorway. In this case, the behaviour ‘causes’ the area choice, resulting in reverse causality. Several studies investigating residential self-selection, however, indicate that regardless of personal preference and intentions on relocation, the built environment still has measurable effects on walking and cycling [36–38].

Finally, it is usually challenging to assess the degree of external validity or generalisability of natural experimental studies. While considerable effort was put into the delineation of the study areas and recruitment, we make no claim that our sample was representative of the local population. Self-report postal surveys may lead to over-representation of more educated and/or less deprived individuals due to the cognitive burden of completion, although compared to other survey methods they may have less social desirability bias [39]. However, it is reasonable to assume that some association between urban roads and local walking and cycling would also be seen in other contexts.

### Comparison with other studies

In the small body of existing literature on the effects of busy or large roads on walking and cycling, a conflicting picture emerges. Although in several studies presence of such roads is correlated with lower walking and/or cycling than in their absence (as expressed in a heterogeneous range of outcomes) [40–45], in some cases the reverse is true [46–48]. In other studies, a mix of both associations was observed or theorised [49–51], and in some cases no association could be seen [20, 29, 52, 53].

In a previous analysis from the parent study from which these data originate, utilising 1 day travel diaries rather than the 7 day data reported here, residents living near the new motorway (M74) were more likely to travel by car than those living further away, but there was no evidence of an association with total active travel (either an increase or a decrease) [20]. In a qualitative exploration from the same study, residents living near the new motorway (M74) described mixed effects of the motorway on active travel [26]. Participants more often described active travel changing experientially rather than in volume or frequency. Taken together with these findings, it is possible that the findings of the present study indicate differing relationships between environment and walking and cycling behaviour depending on whether this behaviour is for utility (active travel) or recreation, and that changes in local behaviour following local changes may be distinct from total travel behaviour.

Aesthetic appeal has been positively associated with recreational walking and cycling [24]. A new motorway could diminish aesthetic appeal, reflecting the lower likelihood of recreational walking and cycling we observed in those living in closer proximity to the new motorway (M74).

Our findings suggested people living near a longstanding motorway (M8) accessed a lower number of types of local destinations by active modes. This could reflect processes of community severance, such as fewer community amenities to access nearer a motorway, or a process by which an individual may be unaware of, or not drawn to local places near a motorway as they redefine their neighbourhood into a smaller geographical area [8, 54]. Severance effects have been described as taking time to accrue [55], which might explain why this association was not observed in the new motorway (M74) study area.

Conversely, our findings also demonstrate a positive association between living near the new motorway (M74) and local active travel, and between road network proximity to a motorway access point and local active travel in those living near the longstanding motorway (M8). It is possible that this reflects some of the beneficial effects of motorways proposed by the advocates of the new M74 – namely that motorways divert what would otherwise be local traffic, making the local area more appealing for walking and cycling [21]. However, as proposed in the previous section, this may also reflect the confounding effect of socioeconomic status, whereby those economically obliged to live nearer the motorway are also less likely to own a car.

### Implications for research

This cross-sectional study provides a justification for further investigation of the association between major urban road infrastructure and local walking and cycling. Establishing the nature, direction and temporality of the associations found in our study will be important to guiding future policy decisions on road construction and planning. We have highlighted a number of potential mechanisms of effect, warranting further study. Our study adds evidence to the theory that recreational walking and cycling, as opposed to active travel, should be considered separately in research and their differing determinants elucidated. We suggest that further research should explore these themes using longitudinal or evaluative methods and detailed assessments of travel behaviour to strengthen the evidence base. This would ideally include more granular assessment of count of trips to different destinations, access to destinations and the diversity of specific destinations accessed.

### Implications for policy

Current national and regional transport policy in Scotland prioritises investment in sustainable transport modes and improving the accessibility of the transport network to disadvantaged communities [56, 57]. In this context, this study offers important quantitative evidence that the association between new roads and walking and cycling is not null, but the direction of effect and explanations for it may be complex. Health impact assessments for new roads should consider potential impacts on total and local walking and cycling.

It is often argued that new roads help regenerate disadvantaged areas [21], suggesting socioeconomic factors should also be considered in any impact assessment. That increased proximity to a new motorway was associated with increased likelihood of local active travel may suggest a health benefit, but may also reflect socioeconomic factors such as the aggregation of those without car access. A recent analysis of Scottish Household Survey 1 day travel diaries found likelihood of an active journey stage was higher for those living in the most deprived areas than for those in the least deprived [58]. The construction of a motorway, intended to facilitate motorised transport, in an area where nearly half the local population do not own a car (44% in our study participants), and therefore cannot make use of the infrastructure, may be contrary to the principles of social justice. However, if socioeconomic circumstances improve as result of a new road, this might have an unintended consequence of increasing car ownership and thus diminishing active travel. How to preserve active travel through such changes is an important public health consideration.

## Conclusion

This cross-sectional study provides quantitative evidence that there is an association between living near an urban motorway and local walking and cycling behaviour. However, the association was complex, suggesting that the age of the motorway and the nature of walking and cycling - for recreation or transport – affected the strength and direction of association.

## Data Availability

Study meta-data are fully available without restriction at http://epi-meta.medschl.cam.ac.uk. Non-identifiable individual-level data are available on request. The MRC Epidemiology Unit Senior Data Manager, Adam Dickinson, manages the processing of data requests (datasharing@mrc-epid.cam.ac.uk). Requests for data sharing will be considered by the principal investigator (DO) in consultation with the other investigators. Their consent prevents the authors from making these data available publicly; third-party researchers would need to sign a collaborative agreement. The authors’ data sharing policies and processes meet the requirements and expectations of MRC policy on sharing of data from population and patient cohorts: http://www.mrc.ac.uk/research/research-policy-ethics/data-sharing/policy/. These policies and processes are in place to ensure that the use of data from this study is within the bounds of consent given previously by study members, complies with MRC guidance on ethics and research governance, and meets rigorous MRC data security standards.

## Abbreviations

CI: Confidence interval
GIS: Geographical Information System
IQR: Interquartile range
OR: Odds ratio
SD: Standard deviation
WHO: World Health Organization
